# State of the Art: trxG Factor Regulation of Post-embryonic Plant Development

**DOI:** 10.3389/fpls.2017.01925

**Published:** 2017-11-14

**Authors:** Jennifer C. Fletcher

**Affiliations:** ^1^Plant Gene Expression Center, United States Department of Agriculture – Agricultural Research Service, Albany, CA, United States; ^2^Department of Plant and Microbial Biology, University of California, Berkeley, Berkeley, CA, United States

**Keywords:** trxG, PcG, development, chromatin, histone methylation, transcription, epigenetics, Arabidopsis

## Abstract

Multicellular organisms rely on the precise and consistent regulation of gene expression to direct their development in tissue- and cell-type specific patterns. This regulatory activity involves arrays of DNA-binding transcription factors and epigenetic factors that modify chromatin structure. Among the chromatin modifiers, trithorax (trxG) and Polycomb (PcG) group proteins play important roles in orchestrating the stable activation and repression of gene expression, respectively. These proteins have generally antagonistic functions in maintaining cell and tissue homeostasis as well as in mediating widespread transcriptional reprogramming during developmental transitions. Plants utilize multiple trxG factors to regulate gene transcription as they modulate their development in response to both endogenous and environmental cues. Here, I will discuss the roles of trxG factors and their associated proteins in post-embryonic plant development.

## Introduction

The development of multicellular organisms is driven by precise patterns of gene transcription that are tightly regulated in a spatial and temporal manner. Establishing and sustaining specific transcription states at gene loci are complex, multi-step processes. They require repertoires of sequence-specific DNA-binding transcription factors as well as epigenetic factors that alter chromatin structure and thereby affect accessibility by the transcriptional machinery. Epigenetic regulators classified as trithorax group (trxG) and Polycomb group (PcG) factors are critical for maintaining the stable transcription patterns at developmental regulatory loci by organizing chromatin in an active or inactive state, respectively (Schwartz and Pirrota, 2007). trxG and PcG factors generally act in large, multi-component complexes that function antagonistically to generate and maintain a balanced state of gene expression (Piunti and Shilatifard, 2016).

TrxG genes were first identified in *Drosophila* as positive regulators of PcG developmental target genes (Ingham, 1988; Kennison, 1995), and their protein products operate in multiple complexes that affect gene expression on a global scale (Piunti and Shilatifard, 2016). Because transcription activation involves numerous steps, trxG factors are heterogeneous and fall into several functional categories: chromatin remodeling proteins, histone modifying methyltransferase and demethylase proteins, and DNA-binding and accessory proteins (Xiao et al., 2016) (**Table 1**). The chromatin remodeling proteins include members of the SWI/SNF, ISWI, and CHD families that utilize ATP to alter nucleosome assembly and distribution (Gentry and Hennig, 2014). The histone modifying enzymes deposit H3K4me2/3 and/or H3K36me2/3 marks associated with transcription activation to counteract the activity of PcG complexes such as POLYCOMB REPRESSIVE COMPLEX 2 (PRC2) that deposit H3K27me3 as the major repressive mark for transcription (Piunti and Shilatifard, 2016).

**Table 1:** Biological functions of the trxG factors and their accessory proteins in post-embryonic development.

trxG factor	Biological function(s)	Reference
Chromatin remodelers		
BRM	Maintains RAM and SAM activity;	Farrona et al., 2004; Yang et al., 2015
	Represses floral transition;	Farrona et al., 2011; Li et al., 2015
	Specifies floral organ identity	Farrona et al., 2004; Wu et al., 2012
CHR11	Promotes floral morphogenesis	Smaczniak et al., 2012
CHR17	Promotes floral morphogenesis	Smaczniak et al., 2012
PKL	Maintains RAM activity	Aichinger et al., 2011
SYD	Maintains SAM activity;	Kwon et al., 2005
	Specifies floral organ identity	Wagner and Meyerowitz, 2002; Wu et al., 2012
Histone methyltransferases		
ATX1/SDG27	Maintains RAM activity;	Napsucialy-Mendivil et al., 2014
	Represses floral transition;	Pien et al., 2008
	Specifies floral organ identity	Alvarez-Venegas et al., 2003
ATXR3/SDG2	Maintains RAM activity	Yao et al., 2013
ATXR7/SDG25	Represses floral transition	Berr et al., 2009; Tamada et al., 2009
SDG8	Represses floral transition;	Shafiq et al., 2014; Yang et al., 2014
	Specifies floral organ identity	Grini et al., 2009
SDG26	Promotes floral transition	Berr et al., 2015
SDG701	Promotes floral transition in rice	Liu et al., 2017
SDG708	Promotes floral transition in rice	Liu et al., 2016
AtCOMPASS core components		
ASH2R	Represses floral transition	Jiang et al., 2011
RBL	Represses floral transition	Jiang et al., 2011
WDR5	Represses floral transition	Jiang et al., 2009
Histone demethylases		
ELF6	Represses floral transition	Yang et al., 2016
REF6/JMJ12	Promotes floral transition	Noh et al., 2004; Yang et al., 2016
DNA-binding and accessory proteins		
ALP1	Restricts FM activity	Liang et al., 2015
NF-Y	Promotes floral transition	Hou et al., 2014
SEP3	Specifies floral organ identity	Pelaz et al., 2000
ULT1/2	Restricts SAM activity;	Carles et al., 2004, 2005
	Restricts FM activity	Carles and Fletcher, 2009

Plant trxG factors have been identified either by homology to known trxG factors in animals or by genetic characterization based on their ability to suppress PcG mutant phenotypes. Given their fundamental roles in the epigenetic regulation of gene expression states, mutations in plant trxG genes often cause pleiotrophic developmental phenotypes, including defects in seedling growth, anther and ovule formation, and gametophyte development (Grini et al., 2009; Guo et al., 2010; Carter et al., 2016; Chen et al., 2017). They also play key roles during developmental transitions when widespread gene reprogramming occurs. Here I will summarize our current understanding of trxG protein function in plant meristems, which drive post-embryonic development.

## Role of trxG Factors in Root and Shoot Apical Meristem Maintenance

Plants are sessile organisms that grow continuously and alter their development in response to changes in their environment. Organogenesis occurs throughout the life cycle from specialized structures at the growing shoot and root tips, called apical meristems (Steeves and Sussex, 1989). Both the root and shoot apical meristems (SAMs) contain small reservoirs of stem cells that constantly replenish themselves as well as provide progeny cells for continuous organ formation. The flexible regulation of gene expression via chromatin remodeling is essential for maintaining these pluripotent stem cell populations whose progeny can assume different fates. Animal stem cells possess special chromatin signatures (Bernstein et al., 2006) that permit plasticity in stem cell dynamics, and much is known about the epigenetic factors and mechanisms involved (Spivakov and Fisher, 2007). In contrast, the roles of epigenetic factors in regulating plant stem cell activity are only beginning to be revealed.

Recent genetic studies have uncovered roles for multiple trxG factors in root apical meristem (RAM) maintenance. The RAM generates the entire underground root system and has a stereotypical organization. Four rarely dividing cells known as the quiescent center (QC) act as a niche (van den Berg et al., 1997) that maintains the surrounding cells as stem cells, aka initial cells, which undergo asymmetric cell divisions to generate the distinct root cell lineages. A gradient of the hormone auxin across the root tip exists due to the activity of members of the PIN-FORMED (PIN) family of auxin transport proteins (Blilou et al., 2005; Grieneisen et al., 2007). The auxin concentration maximum coincides with the QC and promotes the expression of the *PLETHORA (PLT)* AP2 domain transcription factor (TF) genes, which are essential for root stem cell niche maintenance (Aida et al., 2004).

**FIGURE 1:**
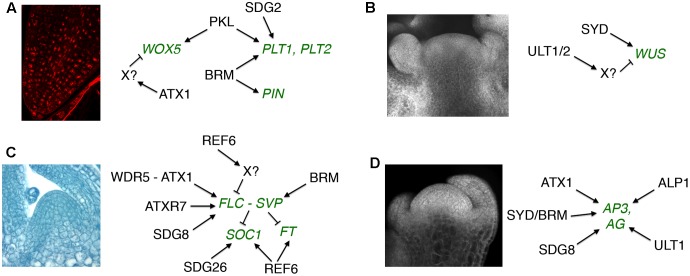
Regulatory targets of trxG factors in post-embryonic development. **(A)** Root apical meristem maintenance. **(B)** Shoot apical meristem maintenance. **(C)** Vegetative to reproductive meristem transition. **(D)** Floral meristem patterning. Gene targets shown in green type. Arrows indicate positive and bars indicate negative regulatory interactions. The SAM image in **(B)** is reprinted from Fiume et al. (2010). No permission is required for its reproduction.

Two H3K4 histone methyltransferase trxG factors have been implicated in Arabidopsis RAM maintenance (**Figure 1A**). The SET domain protein SET DOMAIN GROUP 2 (SDG2) is the major H3K4 trimethyltransferase in Arabidopsis and is necessary for genome-wide H3K4me3 deposition (Guo et al., 2010). In the RAM *SDG2* is required to maintain the auxin gradient and QC maximum, and to sustain cell identity and stem cell activity in the QC and surrounding initial cells (Yao et al., 2013). These functions correlate with a requirement for SDG2 to promote *PLT1* expression and global H3K4me3 deposition in root cells (Yao et al., 2013). The ARABIDOPSIS HOMOLOG OF TRITHORAX1 (ATX1/SDG27) protein contributes ∼15% of genome-wide H3K4 trimethylation (Alvarez-Venegas and Avramova, 2005). ATX1 is needed for TATA binding protein (TBP) and RNA Polymerase II recruitment to its target promoters (Ding et al., 2011) and is also critical for H3K4me3 deposition associated with transcription elongation (Ding et al., 2012). Like SDG2, *ATX1* is necessary for normal RAM organization, but also restricts the expression of QC markers such as *WOX5* to the stem cell niche in an auxin-independent fashion (Napsucialy-Mendivil et al., 2014), indicating that the two H3K4 histone methyltransferases have distinct as well as shared roles in RAM maintenance.

The SWI2/SNF2 chromatin remodeling complex ATPase genes *PICKLE (PKL)* and *BRAHMA (BRM)* also regulate RAM activity in Arabidopsis. PKL acts antagonistically to the PRC2 PcG factor CURLY LEAF (CLF) to maintain RAM stem cell activity (Aichinger et al., 2011). PKL does not induce the activity of the root stem cell niche by affecting auxin accumulation. Instead, PKL elevates the expression levels of and limits CLF-mediated H3K27me3 deposition at *PLT1, PLT2*, and *WOX5* (Aichinger et al., 2011). BRM likewise maintains the RAM stem cell niche by promoting expression of *PLT1* and *PLT2* (Yang et al., 2015). However, BRM unlike PKL affects auxin accumulation in the root tip by directly binding to and up-regulating the transcription of five *PIN* loci (Yang et al., 2015). Thus the evidence to date suggests that the auxin-dependent and auxin-independent regulatory pathways utilize distinct trxG factors to sustain RAM activity.

Maintenance of stem cell reservoirs in Arabidopsis shoot and floral meristems occurs via a spatial negative feedback loop mediated by the *WUSCHEL (WUS)*-*CLAVATA (CLV)* signal transduction pathway. The homeobox TF gene *WUS* is expressed in the core of the meristem and confers stem cell identity on the overlying cells (Laux et al., 1996). The stem cell-inducing activity of *WUS* is antagonized by the secreted polypeptide CLV3 (Fletcher et al., 1999; Rojo et al., 2002), which is produced by the stem cells and activates a signal transduction pathway in the meristem interior that limits the accumulation of *WUS*-expressing cells (Brand et al., 2000). This feedback loop mediates stem cell homeostasis, balancing the loss of stem cells to organ formation with their replenishment via cell division.

Although little is known about trxG activity in the SAM, the ratio of H3K4me3 active to H3K27me3 repressive marks is known to be important for reproductive SAM development in rice (Liu et al., 2015). The SWI2/SNF2 trxG factors BRM and SYD both act to sustain SAM activity in Arabidopsis (Farrona et al., 2004; Kwon et al., 2005), with SYD shown to bind to the *WUS* promoter and elevate its transcription in the SAM (Kwon et al., 2005) (**Figure 1B**). In contrast, the SAND domain-containing proteins ULTRAPETALA1 (ULT1) and ULT2 restrict shoot and floral stem cell activity by limiting the size of the *WUS* expression domain (Carles et al., 2004, 2005). The SAND domain occurs in a small number of eukaryotic proteins including the human AIRE transcriptional regulator that is implicated in autoimmune diseases (Abramson and Goldfarb, 2016). ULT1 antagonizes the repressive activity of PRC1 and PRC2 PcG complex components (Carles and Fletcher, 2009; Pu et al., 2013), and physically associates with the H3K4 methyltransferase ATX1 (Carles and Fletcher, 2009) as well as the transcription factors KANADI1 (KAN1), KAN2 and ULTRAPETALA1 INTERACTING FACTOR (UIF1) (Pires et al., 2014; Moreau et al., 2016). Because both ATX1 and the AIRE protein form complexes with RNA Pol II and RNA-processing components (Abramson et al., 2010; Ding et al., 2012), the ULT proteins may coordinate chromatin mark modification at transcriptionally priming loci with basal transcription machinery recruitment by stage- and tissue-specific transcription factors (Carles and Fletcher, 2010; Engelhorn et al., 2014a).

## Roles of trxG Factors in Floral Induction at the Shoot Apical Meristem

The floral induction process directs the SAM to transition from generating vegetative organs (leaves) to reproductive organs (flowers). The timing of this dynamic meristem cell fate switch is critical for plant reproductive success and occurs in response to endogenous pathways such as the age, GA and autonomous pathways (APs) as well as environmental cues including photoperiod, vernalization and temperature (Amasino, 2010; Srikanth and Schmid, 2011; Andres and Coupland, 2012). The MADS domain TF FLOWERING LOCUS C (FLC) is the main floral repressor in Arabidopsis seedlings (Michaels and Amasino, 1999) and is a key target of both endogenous and environmental signaling pathways (Sheldon et al., 2000; Michaels and Amasino, 2001). During vegetative development FLC directly represses the transcription of the flowering time integrators *FLOWERING LOCUS T (FT), FLOWERING LOCUS D (FD)* and *SOC1*, which promote the transition to flowering (Michaels and Amasino, 2001). FT protein is produced in the rosette leaves and moves to the SAM, where it interacts with the FD protein. The FT-FD complex then induces the floral transition by activating the expression of TF genes such as *SOC1, LEAFY (LFY)* and *APETALA1 (AP1)*, which confer floral meristem (FM) identity on the primordia that form on the flanks of the primary SAM (Irish, 2010).

**FIGURE 2:**
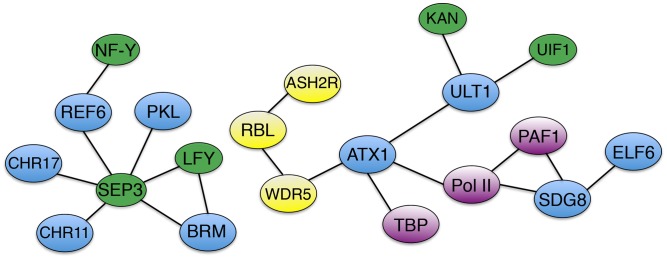
Association networks of trxG factors and interacting proteins. Solid bars designate physical associations between trxG factors (blue), transcription factors (green), AtCOMPASS components (yellow), and transcription machinery components and accessory proteins (purple).

In Arabidopsis, chromatin modifications at key regulatory loci such as *FLC, SOC1* and *FT* are crucial to the timing of the floral transition, and a number of trxG factors are involved in these processes (**Figure 1C**). Because the role of epigenetic factors in *FLC* regulation during vernalization has been extensively reviewed (Jarillo and Pineiro, 2011; Andres and Coupland, 2012; He, 2012; Hepworth and Dean, 2015), I will focus here on the control of flowering through other pathways. To prevent premature flowering during vegetative growth, the *FLC* locus is maintained in a transcriptionally active state marked by H3K36 tri-methylation (Zhao et al., 2005; Yang et al., 2014), which inhibits accumulation of H3K27me3 repressive marks (Shafiq et al., 2014). SDG8 is the major H3K36me3 methyltransferase in Arabidopsis (Yang et al., 2014) and is required for H3K36me3 deposition at the *FLC* locus (Shafiq et al., 2014; Yang et al., 2014). SDG8 associates with components of the transcription machinery, including RNA Pol II and PAF1, as well as with the H3K27 demethylase EARLY FLOWERING 6 (ELF6) (Yang et al., 2016). These physical associations (**Figure 2**) couple removal of repressive histone marks with deposition of active marks and transcription initiation/elongation to sustain high levels of *FLC* expression. ATXR7/SDG25 also represses the floral transition by binding to the *FLC* promoter and augmenting both H3K36me3 and H3K4me3 accumulation (Berr et al., 2009; Tamada et al., 2009), but whether SDG8 and ATXR7 function in concert to induce *FLC* transcription is unknown.

ATXR7/SDG25 does act independently of the H3K4me3 methyltransferase ATX1 in repressing the flowering transition (Tamada et al., 2009), although ATX1 also directly binds to the *FLC* locus to deposit H3K4me3 and elevate expression of the floral repressor gene (Pien et al., 2008). ATX1 physically associates with WDR5a (**Figure 2**), a core component of the Arabidopsis COMPASS-like complex (AtCOMPASS) that also binds the *FLC* locus and promotes its expression by elevating H3K4me3 levels (Jiang et al., 2009). Two other core AtCOMPASS components, ASH2R and RbBP5-LIKE (RBL), also repress the floral transition by enhancing *FLC* expression, with ASH2R availability apparently being the rate-limiting factor in H3K4 tri-methylation at *FLC* and other target loci (Jiang et al., 2011). Therefore both H3K36me3 and H3K4me3 contribute to the maintenance of active *FLC* transcription in Arabidopsis.

The chromatin remodeling ATPase BRM prevents premature flowering by regulating *FLC* and *FLC*-related gene expression (Farrona et al., 2011; Li et al., 2015). Loss of function *brm* seedlings exhibit elevated H3K4me3 levels and reduced H3K27me3 levels in the *FLC* promoter, indicating that BRM imposes a repressive chromatin configuration at the *FLC* locus (Farrona et al., 2011). In addition, BRM directly activates the expression of the *FLC*-related MADS box TF gene *SHORT VEGETATIVE PHASE (SVP)* (Li et al., 2015). SVP forms a complex with FLC to repress flowering under non-inductive conditions (Fujiwara et al., 2008; Li et al., 2008). *SVP* expression is regulated by the AP, GA and temperature pathways, and directly represses *SOC1* and *FT* transcription (Li et al., 2008). BRM represses flowering largely by inducing *SVP* transcription in vegetative tissues, binding to the *SVP* locus where it limits H3K27me3 accumulation by restricting CLF occupancy and activity (Li et al., 2015). Thus the early flowering phenotype of *brm* mutants can be accounted for by a reduction in *SVP* mRNA levels leading to lower abundance of the FLC-SVP repressor complex, resulting in elevated *FT* transcript levels that induce precocious flowering.

Repression of *FLC* transcription is crucial for the transition from the vegetative to the reproductive state. The JmjC domain-containing H3K27 demethylase REF6/JMJ12 promotes flowering independently of photoperiod by repressing *FLC* transcription (Noh et al., 2004). Because REF6 acts antagonistically to CLF by removing repressive H3K27me2/3 marks (Lu et al., 2011), binding its target genes in a sequence-specific fashion via its C2H2 zinc-finger domains (Cui et al., 2016) and facilitating recruitment of BRM (Li et al., 2016), the repression of *FLC* by REF6 is likely to be indirect (Yang et al., 2016). REF6 also induces transcription of the floral activator genes *SOC1* and *FT* in an *FLC*-independent fashion (Noh et al., 2004; Lu et al., 2011). It is recruited by the nuclear factor Y (NF-Y) transcription factor complex to demethylate the *SOC1* locus in response to the photoperiod and GA pathways (Hou et al., 2014), indicating that REF6 is a component of both endogenous and environmental signaling modules. Like REF6, the SDG26 histone methyltransferase also binds to and induces *SOC1* transcription, augmenting the deposition of both H3K4me3 and H3K36me3 at the locus to promote the floral transition (Berr et al., 2015).

Finally, two methyltransferases that accelerate the floral transition independent of photoperiod have recently been characterized in rice. *SDG708* encodes a methyltransferase that deposits up to three methyl groups on H3K36, and promotes flowering by catalyzing H3K36 methylation at the key flowering time regulatory genes *H3Da* and *RFT1*, which are closely related homologs of Arabidopsis *FT*, and *Ehd1* (Liu et al., 2016). *SDG701* encodes an H3K4 di- and tri-methyltransferase that likewise promotes flowering by depositing H3K4me3 to elevate the expression of *H3Da* and *RFT1* (Liu et al., 2017).

## Roles of trxG Factors in Patterning the Floral Meristem

Flowering signals induce reproductive development in plants by reprogramming the SAM into an inflorescence meristem (IM) that produces floral meristems (FMs) instead of leaves. A small suite of floral homeotic transcription factor genes then specifies the identity of each floral organ – sepals, petals, stamens, and carpels – from the outside to the inside of the flower (Coen and Meyerowitz, 1991). The activation of the floral homeotic genes at the onset of flower patterning requires counteracting the PcG-mediated repressive state that has persisted throughout vegetative development (Pu and Sung, 2015), an activity that is associated with increases in H3K4me3 levels at PcG target genes (Engelhorn et al., 2017) and involves multiple trxG factors.

The plant specific TF LFY and the MADS domain TF SEPALLATA3 (SEP3) play crucial roles in activating the expression of MADS box-containing floral homeotic genes such as *APETALA3 (AP3)* and *AGAMOUS (AG)* that specify petal, stamen, and carpel identity in the developing flower (Weigel and Meyerowitz, 1993; Pelaz et al., 2000). SYD and BRM physically associate with the LFY and SEP3 proteins (**Figure 2**), which recruit SYD to the *AP3* and *AG* loci (Wu et al., 2012). At the onset of flower patterning, SYD and BRM redundantly regulate floral organ identity specification (Wagner and Meyerowitz, 2002; Farrona et al., 2004) (**Figure 1D**) by activating *AP3* and *AG* expression, antagonizing CLF activity at the two loci by reducing H3K27me3 deposition and enhancing H3K4me3 deposition (Wu et al., 2012). ATX1 and SDG8 also specify floral organ identity by maintaining floral homeotic gene expression levels (Alvarez-Venegas et al., 2003; Grini et al., 2009), although the mechanistic details are as yet unknown. Finally, REF6 and PKL as well as two ISWI-type chromatin remodelers, CHR11 and CHR17, occur in floral MADS domain protein complexes and affect floral organ morphogenesis (Smaczniak et al., 2012). SEP3 and several other floral homeotic TFs bind their target genes prior to detectable increases in DNA accessibility (Pajaro et al., 2014), suggesting that they work closely with epigenetic factors to facilitate transcription initiation during early flower development by modulating chromatin accessibility at target loci.

The ULT1 trxG protein induces *AG* transcription in the center of the FM at stage 3 of flower formation (Alvarez-Venegas et al., 2003; Carles and Fletcher, 2009), binding directly to the locus to limit CLF-mediated H3K27me3 deposition and enhance H3K4me3 deposition (Carles and Fletcher, 2009). ULT1 thus sets the timing of the transition of the *AG* locus from a repressed to an active state, helping trigger a molecular pathway that ultimately terminates FM activity (Carles and Fletcher, 2009; Engelhorn et al., 2014b; Cao et al., 2015; Sun and Ito, 2015). Mutations in the domesticated transposase gene *ANTAGONIST OF LIKE HETEROCHROMATIN PROTEIN1 (ALP1)* enhance *ult1* FM phenotypes, and *ALP1* promotes floral organ identity gene expression in the absence of LFY (Liang et al., 2015). *ALP1* antagonizes *CLF* function, acting genetically as a trxG factor, and is required for the activity of PcG target genes such as *AP3* and *AG*. Notably, the ALP1 protein complex lacks known trxG factors but consists of core components of PRC2 and accessory factors such as EMF1 and LHP1. ALP1 is therefore proposed to antagonize PcG activity by blocking the interaction between PRC2 and accessory factors that stimulate its activity (Liang et al., 2015).

## Conclusion

A variety of trxG factors exist in plants that carry out diverse biochemical activities to promote active gene expression states: chromatin-remodeling ATPases, histone methyltransferases, AtCOMPASS core components, histone demethylases, as well as DNA-binding and accessory proteins (**Table 1**). It is becoming clear from recent research that members of all of these categories of proteins play important roles in regulating landmark post-embryonic developmental processes such as meristem maintenance and floral induction. Moreover some trxG factors, such as BRM and ATX1, mediate multiple developmental processes during plant growth whereas others appear to have more restricted roles. The coupling of a core set of reiteratively used trxG components together with stage-, tissue- or process-specific trxG components may provide a flexible mechanism for tailoring the basic process of transcription activation to discrete gene networks in response to changing endogenous and environmental signals during the life cycle.

Although significant progress has been made in determining the roles of trxG factors in plant development, many gaps in our understanding remain. It still remains to be determined how many trxG complexes exist in plants, not to mention their full composition and whether that composition is static or changes depending on the developmental stage or tissue. The DNA binding proteins that recruit trxG factors to developmental regulatory loci are only beginning to be identified, while elucidating the chromatin signatures of plant stem cell populations can provide a valuable starting point for determining how tissue- and stage-specific epigenetic states are ultimately achieved during development. Finally, much work remains to decipher how developmental switches between trxG and PcG activities are implemented at individual loci as well as broadly across the genome to coordinate widespread transcriptional reprogramming. Further investigation in these areas will provide a more complete picture of how plants are able to maintain and as necessary adjust their gene expression programs during development in response to a wealth of endogenous and environmental cues.

## Author Contributions

The author confirms being the sole contributor of this work and approved it for publication.

## Conflict of Interest Statement

The author declares that the research was conducted in the absence of any commercial or financial relationships that could be construed as a potential conflict of interest.
